# A specific insertion of a solo-LTR characterizes the Y-chromosome of *Bryonia dioica *(Cucurbitaceae)

**DOI:** 10.1186/1756-0500-3-166

**Published:** 2010-06-14

**Authors:** Ryan K Oyama, Martina V Silber, Susanne S Renner

**Affiliations:** 1 Systematic Botany & Mycology, Ludwig-Maximilians-Universität (LMU Munich), 80638 Munich, Germany

## Abstract

**Background:**

Relatively few species of flowering plants are dioecious and even fewer are known to have sex chromosomes. Current theory posits that homomorphic sex chromosomes, such as found in *Bryonia dioica *(Cucurbitaceae), offer insight into the early stages in the evolution of sex chromosomes from autosomes. Little is known about these early steps, but an accumulation of transposable element sequences has been observed on the Y-chromosomes of some species with heteromorphic sex chromosomes. Recombination, by which transposable elements are removed, is suppressed on at least part of the emerging Y-chromosome, and this may explain the correlation between the emergence of sex chromosomes and transposable element enrichment.

**Findings:**

We sequenced 2321 bp of the Y-chromosome in *Bryonia dioica *that flank a male-linked marker, *BdY1*, reported previously. Within this region, which should be suppressed for recombination, we observed a solo-LTR nested in a *Copia*-like transposable element. We also found other, presumably paralogous, solo-LTRs in a consensus sequence of the underlying *Copia*-like transposable element.

**Conclusions:**

Given that solo-LTRs arise via recombination events, it is noteworthy that we find one in a genomic region where recombination should be suppressed. Although the solo-LTR could have arisen before recombination was suppressed, creating the male-linked marker *BdY1*, our previous study on *B. dioica *suggested that *BdY1 *may not lie in the recombination-suppressed region of the Y-chromosome in all populations. Presence of a solo-LTR near *BdY1 *therefore fits with the observed correlation between retrotransposon accumulation and the suppression of recombination early in the evolution of sex chromosomes. These findings further suggest that the homomorphic sex chromosomes of *B. dioica*, the first organism for which genetic XY sex-determination was inferred, are evolutionarily young and offer reference information for comparative studies of other plant sex chromosomes.

## Background

The origin and evolution of sex chromosomes from autosomes have long interested biologists. Dioecy, a precondition for sex chromosomes where the male and female functions are separated onto different individuals, is common in animals but relatively rare among flowering plants, occurring in only about 6% of species [1]. Sex chromosomes in flowering plants are even less common, with only a handful of species known to have them [2,3]. Molecular clock dating and the observation that some of these species (*e.g*., papaya) still have homomorphic gonosomes, suggest that plant sex chromosomes are evolutionarily young [4,5].

Most of what we know about the evolution of sex chromosomes in flowering plants is derived from research on a few model systems. Molecular level investigations in *Rumex*, *Silene *and *Carica *have yielded a wealth of data [6]. In *Fragaria*, there is evidence of at least some recombination between the sex-determining loci, a hallmark of incipient sex chromosomes, offering support for models of the gradual evolution of sex chromosomes [4,7,8]. Nevertheless, the early steps in the evolution of sex chromosomes from autosomes remain unclear as direct evidence is rare [9]. An empirical correlation observed is the accumulation of transposable elements and other repetitive sequences on the Y- (or W-) chromosome [10].

Transposable elements are ubiquitous components of all genomes. In angiosperms, they make up a particularly large component of genomes and are mostly long-terminal repeat (LTR) retrotransposons in the *Copia *or *Gypsy *superfamilies [11]. Like all retrotransposons, LTR-retrotransposons move intracellularly by a replicative mechanism similar to that of retroviruses and transpose through reverse transcription of an RNA intermediate. LTR-retrotransposons in particular tend to transpose into other LTR-retrotransposons [12-14]. Removal of retrotransposon DNA from the plant genome is thought to require recombination [15]. One such mechanism for removal, unequal homologous recombination, involves recombination between the two LTRs of an LTR-retrotransposon resulting in a solo-LTR [16], making the presence of a solo-LTR a signal of recombination. Conversely, one would expect accumulation of retrotranspon sequences, but not solo-LTRs, where recombination is suppressed, such as on the Y-chromosome [17]. The emerging picture appears, however, to be more complex.

In *Silene latifolia*, *Copia*-like retrotransposons are not preferentially accumulated on the sex chromosomes, but are enriched on the Y- compared to the X-chromosome [18-20]. In *Rumex acetosa*, specific transposable elements accumulate on the two versions of the Y-chromosome [21,22]. Non-LTR retroelements have been found in the terminal region of the long arm of the Y-chromosome of *Cannabis sativa *[23]. In *Carica*, which has homomorphic sex chromosomes, the male-specific Y-region is differentiated from the corresponding region on the X-chromosome by an accumulation of transposable elements and inverted repeats [24], suggesting that retrotransposon accumulation begins very early in the establishment of sex chromosomes. Here we report findings from *Bryonia dioica *(Cucurbitaceae), a species with homomorphic sex chromosomes, in which we have found a complex association of two LTR-retrotransposons.

The cucurbit *Bryonia dioica *was the first organism for which an XY sex-determination system was inferred from the sex ratios obtained in reciprocal crosses between the dioecious *B. dioica *and the monoecious *B. alba *[25,26]. A phylogeny [27] and biogeographic study [28] of the ten species of *Bryonia *indicate that dioecy may have re-evolved in the lineage leading to *B. dioica*, implying that the homomorphic sex chromosomes are evolutionarily young (*i.e*., a few million years old). In our previous paper [29], we found that the male-linked marker *BdY1 *may not lie in the zone of recombination-suppression on the Y-chromosome in all populations, suggesting a recent origin. A second marker, *BdX1*, was common to both male and females, but was highly similar in sequence to *BdY1*, differing mainly by the presence of a large insertion. In this study we used *BdY1 *as an anchor point to perform chromosome walking along the Y-chromosome of *B. dioica*, which revealed a *Copia*-like element within which a solo-LTR is inserted. We also recovered the consensus sequence of the underlying *Copia*-like retrotransposon via genome walking within which we found solo-LTRs inserted at multiple, presumably paralogous locations, one of which created *BdY1 *and thus characterizes the Y-chromosome.

## Results

We sequenced 415 bp upstream and 1649 bp downstream of the SCAR marker *BdY1 *described in Oyama *et al*. [29] on the Y-chromosome of *Bryonia dioica*. Within this sequence (hereafter CW278-Y), which has been submitted to GenBank [GenBank:HM365927], we found an LTR-retrotransposon. We identified the open reading frame (ORF) and, using specific primers for the coding region, continued sequencing the retrotransposon via genome walking. This consensus sequence of the underlying retrotransposon (hereafter RLC-sequence) had a final consensus length of 5008 bp [GenBank:HM365926].

A BLASTn [30,31] search in the TREP database [32] with CW278-Y found the highest similarity to an element from the *Angela *family of LTR-retrotransposons in the *Copia *superfamily [33], while a BLASTn search in GenBank yielded similarity to a *Copia*-like element from *Cucumis *(Cucurbitaceae) [GenBank:GQ326556]. BLASTx analyses of the translated amino acid sequence from the RLC-sequence in GenBank also found similarity to elements in the *Copia *superfamily. Conserved domain search analysis [34] demonstrated that this transposable element encodes a polyprotein with a CCHC zinc knuckle (*gag*) domain (position 1890-1934 on the RLC-sequence, amino acids 254-268), an integrase core (*rve*) domain (position 2616-3098 on the RLC-sequence, aa 496-656) and a reverse transcriptase (*RVT-2*) domain (position 3600-4361 on the RLC-sequence, aa 823-1077). Because we could not identify a protease domain, we assume that this element is inactive. That the domains within the polyprotein (*pol*) gene are arranged in the order *rve*/*RVT *also supports assignment to the *Copia *superfamily.

**Figure 1:**
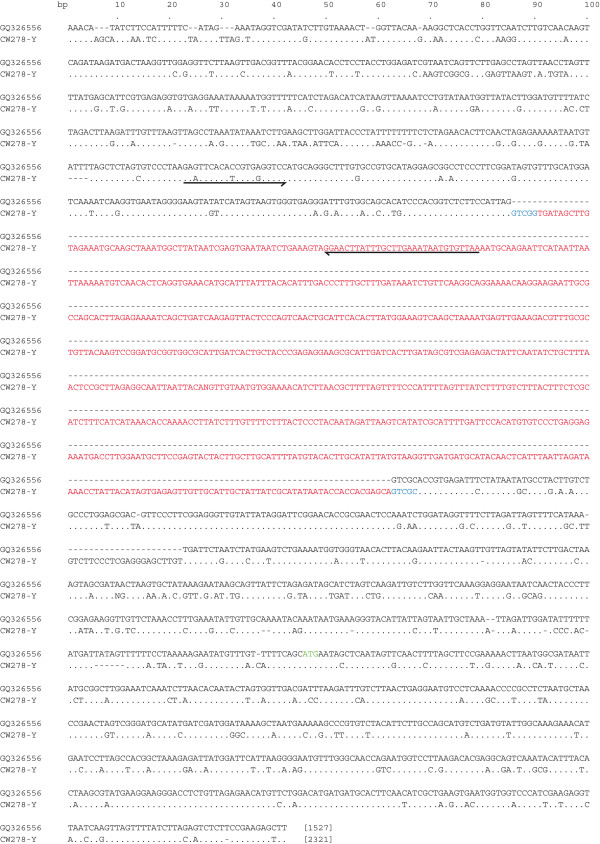
**Alignment of CW278-Y with a sequence of a *Copia*-like retrotransposon from *Cucumis***. The alignment length is 2340 bp and the length of each sequence is given in brackets at the end. The solo-LTR is in red, the flanking repeats are in blue and the start codon of the retrotransposon is in green (at position 2197 of the alignment). The sequence of the element from *Cucumis *(GQ326556) presented here is the reverse complement of the sequence in GenBank.

**Figure 2:**
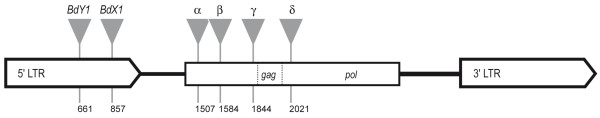
**Locations of the solo-LTRs within the RLC-sequence**. Schematic representation illustrating the locations of the paralogous solo-LTRs. The position numbers at which each insertion begins are in reference to the consensus sequence of RLC_XXX_Bryonia (RLC-sequence), the common *Copia*-like element in which the solo-LTRs are found. The coding region begins at bp 1131.

A putative solo-LTR of 772 bp was identified in CW278-Y beginning at position 584 of the sequence. This was done by aligning the CW278-Y sequence to the *Copia*-like element from *Cucumis *found in BLASTn searches (see Figure 1). The solo-LTR is flanked by 5-bp-long direct but incomplete repeats (GTCGG at the 5'end and GTCG at the 3'end) representing the target-site duplication and contains the 5'-terminal and 3'-terminal sequences 5' TG...CA 3'. This insertion event corresponds to the male-linked SCAR marker *BdY1 *described in Oyama *et al*. [29]. Multiple, presumably paralogous insertion sites within the RLC-sequence could be detected by PCR using internal primers specific to the solo-LTR, the 5'-LTR and the coding region. One of these additional events corresponds to the *BdX1 *sequence described in Oyama *et al*. [29] and the remainder were named α, β, γ and δ (see Figure 2).

**Figure 3:**
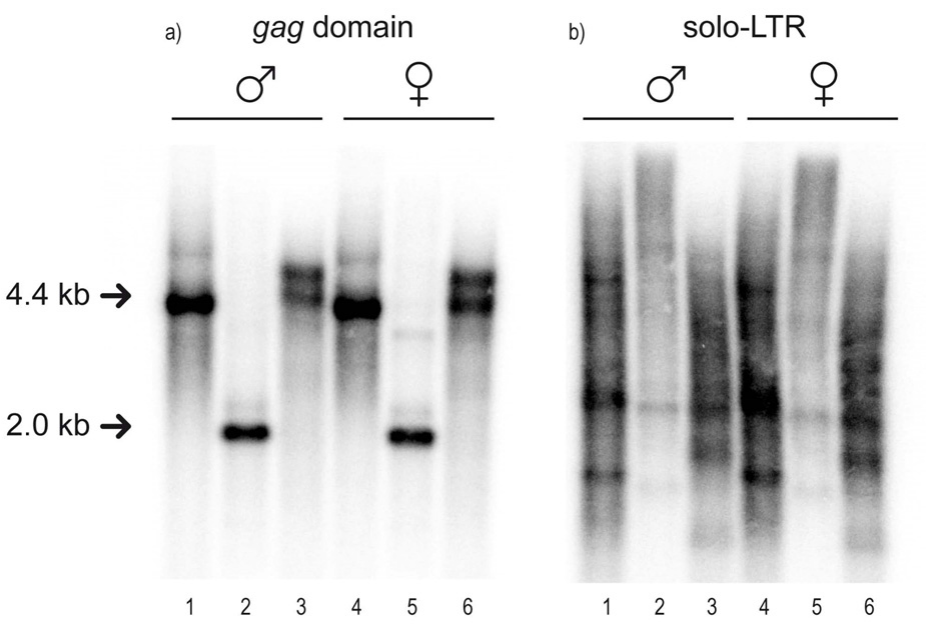
**Southern blots of male and female *Bryonia dioica***. Southern blots using (a) the *gag *domain of the *Copia*-like sequence and (b) the solo-LTR as probes against male (lanes 1-3) and female (lanes 4-6) genomic DNA of *Bryonia dioica*. Restriction enzymes used were *Hind*III (lanes 1 and 4), *EcoR*I (lanes 2 and 5), and *Hae*III (lanes 3 and 6).

Southern blot analysis on male and female plants using 1110 bp of the 5' ORF containing the *Gag *domain as a hybridization probe revealed no significant differences between the two sexes. All digests exhibit strong signals showing the high copy number of these elements in both males and females. Because of *EcoR*I and *Hind*III restriction sites in the 5'-LTR and in the coding region, digestion with these enzymes results in single bands of, respectively, 2 kb and 4.4 kb (Figure 3a). The distinct fragments observed by *Hae*III restriction (Figure 3a) are compatible with the single *Hae*III recognition site in the 5'-LTR revealed by sequencing.

A more complex profile was observed in Southern blots that used the solo-LTR as the probe (Figure 3b). Hybridization patterns in all digests revealed strong smears over the whole lane rather than strong bands, suggesting the existence of many copies of the solo-LTR or the corresponding transposable element in random positions in the genome. However, some digests, such as *Hind*III, show a degree of conservation of this LTR family by the presence of a few, more intense bands of varying sizes. These results are consistent with results from rice using Southern blots, which found some elements to exist in relatively low copy numbers [35,36], and with the observation that LTR retrotransposons are otherwise highly represented in the genome of plants [11].

## Discussion

The first male-linked molecular marker in *Bryonia dioica*, reported by Oyama *et al*. [29], is the result of the insertion of one LTR-retrotransposon into another and the subsequent conversion of the inserted element into a solo-LTR. In our earlier paper, a second sequence was also recovered from both males and females that appeared to have an insert relative to the male-linked marker *BdY1 *but was otherwise alignable. This second sequence appears to result from a paralogous insertion of the element that gave rise to the solo-LTR about 200 bp downstream of the male-linked one. Since the reverse SCAR primer lies inside the solo-LTR, the PCR primer-binding site is effectively moved 200 bp downstream, giving rise to the second PCR band.

A minimum of four other, presumably paralogous, solo-LTRs appear in the underlying retrotransposon sequence. Such multi-layered insertion events have also been observed in maize [14] and probably result from the tendency of LTR-retrotransposons to insert into other LTR-retrotransposons [37]. Since solo-LTRs are the remnants of intact LTR-retrotransposons that were removed via unequal homologous recombination, this means that the underlying *Copia*-like retrotransposon is older than the solo-LTR. Considering that transposable elements are silenced and begin to accumulate mutations quickly [24], one probable reason why we cannot identify the proteinase domain of the *Copia*-like element is that the necessary motif is too degraded.

Methylation is thought to be important in regulating both the activity of transposable elements and recombination. Most of the transposable elements in plant genomes have been heavily methylated, and this is thought to be the mechanism by which the host genome deactivates the transposons [24]. At the same time, methylation is known to suppress recombination [38], the mechanism by which transposable elments would otherwise be eliminated from the genome. With respect to the evolution of sex chromosomes, the suggestion has been made that the accumulation of transposable elements, through their tendency to become methylated, may be what leads to the initial suppression of recombination and allows for the linkage of sex-determining genes [39]. The suppression of recombination would also prevent the removal of transposon sequences, causing the non-recombining gonosome (*e.g*., the Y-chromosome) to appear to accumulate transposable element sequences.

Stress is known to be an activator of LTR-retrotransposons [24] and may relate to the repeated shifts between monoecy and dioecy among the ten species of *Bryonia *[27]. The range expansion of *B. dioica *into northern Europe following the last glacial maximum [28] could have been associated with cold stress and enhanced transposon activity. Population genetic analyses of the male-linked marker *BdY1 *throughout northern and southern Europe seemed to detect ongoing recombination in southern Europe [29]. It could be, however, that the recombination detected is actually a signal of transposition, given that the region flanking *BdY1 *is a hybrid of the underlying *Copia*-like element and the inserted solo-LTR.

## Conclusions

The interesting aspect of the solo-LTR indel event described here is that it is male-linked and located in the non-recombining region of the Y-chromosome. That our walk along the Y-chromosome recovered sequence of a *Copia*-like retrotransposon is congruent with the prevalence of this element in the plant genome, and nesting of LTR-retrotransposons within each other has also been seen previously [14]. However, in light of current theory about sex chromosome evolution, these results may help illuminate the early steps from autosome to sex chromosome; they imply that the recombination event leading to the solo-LTR and the suppression of recombination that would allow this area to be a male-linked marker occurred close together in evolutionary time. Whether the two events are merely correlated or causally linked remains to be clarified. Nevertheless, that a solo-LTR, formed by recombination, is male-linked and characterizes the Y-chromosome, where recombination should be suppressed, provides an interesting insight into the early stages of the evolution of sex chromosomes.

## Methods

### Development of male-linked SCAR marker

The development of the original male-linked SCAR marker (*BdY1*) that served as the anchor point for the chromosome walking was described in Oyama *et al*. [29] and is only summarized here. An AFLP library was created for each sex of *Bryonia dioica *that pooled DNA from eight individuals. This library was filtered and screened for male-linked bands. Putative male-linked bands were confirmed by repeating the procedure for several individuals of each sex. These male-linked bands were isolated and sequenced. SCAR primers were designed from the AFLP sequence, and PCR was performed using these primers and genomic DNA from multiple male and female individuals of *B. dioica *and other *Bryonia *species.

### Sequencing the 5' and 3' regions flanking *BdY1*

DNA was extracted from young *Bryonia dioica *male and female leaves following the cetyltrimethylammonium bromide (CTAB) based method [40]. To determine 5'- and 3'-flanking sequences of *BdY1*, a library was constructed with the GenomeWalker Universal Kit (Clontech Laboratories, Saint-Germain-en-Laye, France) according to the manufacturer's protocol. Briefly, genomic DNA was digested with *EcoR*V, *Dra*I and *Ssp*I and DNA fragments were ligated with a GenomeWalker adaptor supplied in the kit. This GenomeWalker library was used as a template for PCR with an adaptor primer and a gene-specific primer. The PCR mixture was diluted and used as a template for a secondary or "nested" PCR with the nested adaptor primer and a nested gene-specific primer. Cycle conditions for the first PCR: 1 cycle of 3 min at 94°C, 39 cycles of 30 s at 94°C, 1 min at 59°C and 3 min at 68°C for amplification, and 1 cycle of 10 min at 68 °C for final extension. Cycle conditions for the second PCR: 5 cycles of 25 s at 94°C, 3 min at 72°C, 20 cycles of 25 s at 94°C, 3 min at 67°C for amplification and 67°C for 10 min after the final cycle. All PCR mixtures contained 1.25 Units *Taq *DNA polymerase (QIAGEN GmbH, Hilden, Germany [Qiagen]), 1× *Taq *buffer, 15 mM MgCl_2_, 10 mM of each dNTP and 10 pmol of each primer in a total volume of 50 μl. Gene-specific primers (GSP) are listed in Table 1. Amplicons were purified for sequencing and sequencing reactions were performed with the BigDye Terminator v3.1 cycle sequencing kit (Applied Biosystems, Foster City, California [ABI]). The cycle sequencing products were cleaned by Sephadex G-50 gel filtration (GE Healthcare, Freiburg, Germany) and fragments were separated on an ABI 3130 Genetic Analyser capillary sequencer. Sequences were assembled and edited with Sequencher 4.2 (Gene Codes Cooperation, Ann Arbor, Michigan).

**Table 1:** Primer sequences used for chromosome and genome walking

Primer sequences for chromosome walking on the Y-chromosome	
GSP7	5'-TGCATTTCTACAAGCTATCACCGACC
GSP8	5'-AGAGACCGTGGGACATGGTGTCACATACCTC
GSP16	5'-GCCGTGCATAGGAGCAGACTC
GSP17	5'-AGTGGGTGAGAGGTATGTGACACCATGTC
GSP18	5'-ACGGTCTCTTCCATTAGGTCGGTGATAGC
GSP20	5'-AAGGAAGAATTGCGCCAGCAC
GSP21	5'-CAGCTGATCAAGAGTTACTCCCAGTCAACTG
GSP22	5'-TCCGGATGCGGTGGCGCATTG
GSP23	5'-AATGCGCCACCGCATCCGGAC
GSP24	5'-TCAGGGACCATGACGGTTGC

Primer sequences for genome walking to recover retrotransposon sequence	

GSP15	5'-GGTCTCTTCCATTAGGTCGCACCGTGAG
GSP17	5'-AGTGGGTGAGAGGTATGTGACACCATGTC
GSP19	5'-ACTACCCTTCGGTAGATGTTGC
GSP19-1	5'-TGCAATCAAGACGGGCATTGG
GSP19-7	5'-CCTTAGAGCTCGTACATTCGGAC
GSP19-8	5'-CCCAGCAGAACGGTGTATCAG
GSP19-9	5'-TGGATATGGTTCGCTCTATGATGAGC
GSP19-12	5'-ATGGTTCGCTCTATGATGAGC
GSP19-14	5'-GATGGCGTAGAGGATCCACTG
GSP19-15	5'-TGATGTGGATCGTGACCAGTGG

#### Southern Analysis

For Southern analysis, total cellular DNA from *Bryonia dioica *male and female tissues were extracted using the protocol of Fulton *et al*. [41]. Equal amounts of DNA were digested with *Hin*dIII, *EcoR*I and *Hae*III, separated electrophoretically on 1% agarose gels, and transferred to a Biodyne B nylon membrane (Pall Gelman Laboratory, Ann Arbor, Michigan). Hybridizations were performed under high stringency conditions for 24 h at 60°C using [α-^32^P]dCTP-radiolabeled probes. Probes were obtained through PCR amplification using primer pair 5'-GTGCAACTCTTAGCTTCCGAG/5'-TTCGGCTGAGACAACCTCTCC for the *gag *domain and 5'-ACGGTCTCTTCCATTAGGTCGGTGATAGC/5'-AAGAGACCCTATCCATTTCTGGAGTTCG for the solo-LTR. PCRs were carried out under the same conditions as used in the first PCR for genome walking. The products were purified with QIAquick gel extraction kit (Qiagen).

## Competing interests

The authors declare that they have no competing interests.

## Authors' contributions

RKO helped conceive and plan the study, analyzed the data, and drafted the manuscript. MVS gathered the sequence and Southern blot data, analyzed the data, and helped draft the manuscript. SSR conceived of the project and also worked on the manuscript. All authors have read and approved the final manuscript.
